# A systematic review of St. John’s wort for major depressive disorder

**DOI:** 10.1186/s13643-016-0325-2

**Published:** 2016-09-02

**Authors:** Eric A. Apaydin, Alicia R. Maher, Roberta Shanman, Marika S. Booth, Jeremy N. V. Miles, Melony E. Sorbero, Susanne Hempel

**Affiliations:** 1 Pardee RAND Graduate School, RAND Corporation, 1776 Main St, PO Box 2138, Santa Monica, CA 90407-2138 USA; 2 Akasha Center for Integrative Medicine, Santa Monica, CA USA; 3 RAND Corporation, Santa Monica, CA USA

**Keywords:** St. John’s wort, Major depressive disorder, Complementary and alternative medicine, Herb, Systematic review, Meta-analysis, Antidepressant

## Abstract

**Background:**

This systematic review evaluated St. John’s wort (SJW) for the treatment of Major Depressive Disorder (MDD). The objectives of this review are to (1) evaluate the efficacy and safety of SJW in adults with MDD compared to placebo and active comparator and (2) evaluate whether the effects vary by severity of MDD.

**Methods:**

We searched PubMed, CINAHL, PsycINFO, CENTRAL, Embase, AMED, MANTIS, Web of Science, and ICTRP and existing reviews to November 2014. Two independent reviewers screened the citations, abstracted the data, and assessed the risk of bias. We included randomized controlled trials (RCTs) examining the effect of at least a 4-week administration of SJW on depression outcomes against placebo or active comparator in adults with MDD. Risk of bias was assessed using the Cochrane Risk of Bias tool and USPSTF criteria. Quality of evidence (QoE) was assessed using the GRADE approach.

**Results:**

Thirty-five studies examining 6993 patients met inclusion criteria; eight studies evaluated a hypericum extract that combined 0.3 % hypericin and 1–4 % hyperforin. The herb SJW was associated with more treatment responders than placebo (relative risk [RR] 1.53; 95 % confidence interval [CI] 1.19, 1.97; I^2^ 79 %; 18 RCTs; *N* = 2922, moderate QoE; standardized mean differences [SMD] 0.49; CI 0.23, 0.74; 16 RCTs; I^2^ 89 %, *N* = 2888, moderate QoE). Compared to antidepressants, SJW participants were less likely to experience adverse events (OR 0.67; CI 0.56, 0.81; 11 RCTs; moderate QoE) with no difference in treatment effectiveness (RR 1.01; CI 0.90, 1.14; 17 RCTs, I^2^ 52 %, moderate QoE; SMD −0.03; CI −0.21, 0.15; 14 RCTs; I^2^ 74 %; *N* = 2248, moderate QoE) in mild and moderate depression.

**Conclusions:**

SJW monotherapy for mild and moderate depression is superior to placebo in improving depression symptoms and not significantly different from antidepressant medication. However, evidence of heterogeneity and a lack of research on severe depression reduce the quality of the evidence. Adverse events reported in RCTs were comparable to placebo and fewer compared with antidepressants. However, assessments were limited due to poor reporting of adverse events and studies were not designed to assess rare events. Consequently, the findings should be interpreted with caution.

**Systematic review registration:**

PROSPERO CRD42015016406.

**Electronic supplementary material:**

The online version of this article (doi:10.1186/s13643-016-0325-2) contains supplementary material, which is available to authorized users.

## Background

Depressive disorders are one of the largest sources of disease burden. More than 350 million people worldwide suffer from depression at any one time, and this number appears to be on the rise [1]. The condition affected approximately 15 million individuals in the USA in the last year, with a 12-month prevalence of 4.8 % in men and 8.2 % in women, yet the condition remains underdiagnosed and undertreated [2]. Depression has severe consequences for the lives of individuals. Nearly 43 % of those with severe depression in the USA report serious difficulties with work, home, or social activities [3]. Depression is also linked to an estimated productivity loss of 5.6 h per week and $40 billion a year [4].

Pharmacotherapy and psychotherapy are established treatments and have been shown to be effective to treat depressive disorders, such as major depressive disorder (MDD). However, stigma, costs, discomfort with, or lack of availability of, mental health treatment, side effects of medication, and other factors cause many individuals to not seek standard treatments. For centuries, extracts of the herb St. John’s wort (botanical name *Hypericum perforatum* L., SJW) have been used to treat various conditions, including depressive disorders. Existing clinical practice guidelines vary in their recommendations to include SJW as a treatment option for treating depressive disorders [5]. A Cochrane Review of SJW for depression documented available research studies published to 2008 and found a beneficial effect compared to both placebo and other antidepressant therapies across 29 double-blind randomized controlled trials (RCTs) [6]. The review concluded that the available evidence suggested that hypericum extracts tested in the included trials are superior to placebo and patients with major depression and are similarly effective as standard antidepressants, and have fewer side effects than standard antidepressants. Overall, SJW has been considered safe but side effects have been noted, including photosensitivity, elevated thyroid stimulating hormones, hypertensive crisis, and induction of mania [7]. In addition, preparations of SJW vary in the amounts of active compounds they contain, which may make it difficult to compare across studies [8].

In recent years, more research on SJW has been published in the international literature testing not only its effectiveness compared to placebo conditions but testing also its comparative effectiveness and comparative safety compared with standard antidepressant treatment. This review aims to synthesize all available RCTs in a comprehensive systematic review in order to provide reliable and current estimates of the effectiveness and comparative effectiveness and safety of SJW compared to placebo or antidepressant treatment in the treatment of adults with MDD (see Additional file 1 for PRISMA checklist).

We set out to answer the following review questions:

- What are the efficacy and safety of SJW in adults with MDD compared to placebo and active comparator?
- Is there a difference in effect, depending on the type of MDD (i.e., mild, moderate, severe)?

## Methods

### Search strategy

We searched the electronic databases PubMed, CINAHL (Cumulative Index to Nursing and Allied Health Literature), PsycINFO, CENTRAL (Cochrane Central Register of Controlled Trials), Embase, AMED (Allied and Complementary Health Database), MANTIS (Manual, Alternative, and Natural Therapy Index System), Web of Science, and ICTRP (International Clinical Trials Registry Platform) without language restriction from January 2007 to November 2014 to identify recent reports of RCTs testing the efficacy and safety of SJW—used adjunctively or as monotherapy—to treat adults with MDD. RCTs published earlier than 2007 were identified through reference mining of included studies and previous systematic reviews related to SJW, including a Cochrane review that included trials on SJW for MDD published to July 2007 [6]. The Cochrane review conducted a comprehensive search to locate SJW RCTs in the Clinical Trials Register of the Cochrane Collaboration Depression Anxiety & Neurosis Group (CCDANTR) until 2007, in PubMed until 2008, in the database of the Cochrane Field for Complementary Medicine, in the Medline SilverPlatter CD-ROM from 1983 onwards, in Embase from 1989 onward, in the Psychlit and Psychindex 1987–1997 CD-ROM, and in Phytodok [6]. We screened all studies identified in the systematic searches, i.e., studies included or excluded from the Cochrane review. All studies included in the 2008 Cochrane review were eligible for inclusion, but our review also identified head-to-head trials comparing different St. John’s wort extracts, different dosage, and standard antidepressant interventions (including psychotherapy). Our search was not limited to peer-reviewed literature; we included grey literature, such as conference abstracts. We contacted authors to obtain full-text publications cited in other reviews or indexed in databases that were not available through information retrieval services or the original publisher; but, due to resource restrains, we did not systematically contact all authors for potential additional studies or data. The search strategy is available online. (see Additional file 2).

### Eligibility criteria

The inclusion and exclusion criteria for this review were developed using the framework of participants, interventions, comparators, outcomes, timing, settings, and study design or PICOTSS:

- *Participants*: Studies in adults, male and female, 18 years of age and over, with a diagnosis of MDD were eligible for inclusion in the review. In studies not referring to a clinical diagnosis based on *Diagnostic and Statistical Manual of Mental Disorders* (DSM) or International Classification of Diseases (ICD) criteria, we applied a specified threshold on validated depression scales (see Additional file 3). Studies that enrolled individuals with other comorbid conditions, such as traumatic brain injury, were eligible for inclusion. Studies in participants in postnatal depression were included if the criteria were in accordance with DSM-V criteria for MDD (i.e., peripartum onset or 4 weeks following delivery). Studies in individuals with diagnoses of dysthymia, bipolar disorder, or schizophrenia, alone or in combination with major depression, were excluded in accordance with DSM-V criteria. Studies evaluating multiple psychiatric conditions were included if the data for patients with MDD were presented separately.
- *Interventions*: Studies that administered a supplement that contained a known amount of SJW, and the amount and type of active compounds contained in the SJW supplement that was specified (i.e., naphthodianthrones, hypericin, pseudohypericin, flavonoids, phloroglucinols, hyperforin, and adhyperforin), were eligible. SJW could be evaluated alone or in conjunction with pharmacologic and/or psychotherapy.
- *Comparator*: Studies comparing SJW with placebo or with active comparators, or against another amount or extract of SJW, were eligible.
- *Outcomes*: Studies that reported Hamilton clinical rating scale for depression (HAMD) scores or other validated depression scale scores were eligible for inclusion as well as studies that reported other changes in depressive symptoms (e.g., suicidal ideation) or the rate of treatment responders. Studies that reported the number of patients in remission or rates of depression relapse were also eligible. Studies that reported adverse events in adults taking SJW for MDD were included if adverse events were reported by study arm. Studies that reported on biomarkers alone without reporting efficacy for depression outcomes were excluded. Only studies that at least reported outcome assessments at baseline and at the end of treatment for both study arms were included. Studies of healthcare provider outcomes, acceptance, prevalence, use, costs, study design features, and intervention features not reporting patient health outcomes were excluded.
- *Timing*: Only studies with a treatment duration of 4 weeks or longer were eligible.
- *Setting*: Studies were not limited by setting (e.g., country, physical location of treatment).
- *Study design*: Included studies were limited to RCTs.

### Inclusion screening

All article screening and abstraction was conducted using the systematic review software DistillerSR (Evidence Partners, Ottawa, Canada). Two independent reviewers screened titles and abstracts of retrieved citations. Citations judged as potentially eligible by one or both reviewers were obtained as full text. The full-text publications were screened against the specified inclusion criteria by the two independent reviewers using a standardized and pilot-tested form; any disagreements were resolved through discussion within the review team.

Studies reporting on the same participants were counted as one study regardless of the number of publications the results were presented in. All study-related publications were considered and contributed to the data extraction.

### Data extraction

Two reviewers abstracted study-level information. Categorical data concerning study details were abstracted independently by both reviewers; free text information concerning study details were abstracted by one reviewer and checked by the review lead. The reviewers pilot-tested the data collection forms prior to data extraction to ensure agreement of interpretation. Numerical outcome data were abstracted and checked by a single biostatistician.

The following information was abstracted from each study:

- *Participants*: MDD diagnostic criteria, baseline measure of depression symptoms, depression severity (mild, moderate, or severe) using the authors’ description, depression history (e.g., recurrent), comorbidities, mean age and age range, gender
- *Interventions*: details including amount and type of active compounds contained in the SJW supplement, dosage, co-intervention(s)
- *Comparators*: type and description of comparator
- *Outcomes assessed*: assessment measures and primary endpoint, method of data expression (e.g., mean difference), results (effect estimate, precision)
- *Timing*: time-points of outcome assessment, duration of intervention
- *Setting*: country
- *Study design*: aim of study, inclusion and exclusion criteria, sample size and reported power calculations, funding source.

Outcome data were based on intention-to-treat (ITT) analyses. In the absence of reported ITT data, we used the number randomized as the denominator; in the absence of the number randomized, we used the number of participants at follow-up. All studies were analyzed using the latest reported follow-up; however, studies reporting follow-up only for a subsample of treatment responders were not considered. Follow-up used the baseline as the point of reference, not the end of treatment; most studies assessed treatment effects directly after the end of treatment but treatment duration varied. When multiple depression measures were available, we used HAMD scores to assess treatment effects on depression symptoms. We used the authors’ definition of response to treatment, usually reflecting a 50 % decrease in HAMD scores. We used the authors’ definition of remission, usually reflecting a HAMD score of less than seven or eight. We computed standardized mean differences (SMDs) for studies reporting continuous outcomes, relative risks (RRs) for treatment effect estimates, and odds ratios (ORs) for rare adverse events, together with the 95 % confidence interval (CI).

### Risk of bias

Two reviewers independently assessed the risk of bias of included studies using the Cochrane Risk of Bias tool [9] and criteria used by the US Preventative Services Task Force [10]. We assessed random sequence generation (selection bias); allocation concealment (selection bias); blinding of participants and providers (performance bias); blinding of outcome assessors (detection bias); completeness of reporting outcome data (attrition bias); selective outcome reporting (reporting bias); whether treatment group received plus treatment as usual SJW and the control group received treatment as usual plus no additional treatment (“add-on trial”); washout periods or exclusion of individuals taking personal supplement; equal distribution among groups of potential confounders at baseline; crossovers or contamination between groups; equal, reliable, and valid outcome measurement; clear definitions of interventions; and ITT analysis. The criteria were used to rate the quality of individual studies using the following guidelines [10, 11]:

- *Good*: Comparable groups are initially assembled and maintained throughout the study with at least 80 % follow-up; reliable, valid measurement is used and applied equally to all groups; interventions are clearly described; all important outcomes are considered; appropriate attention is given to confounders in analysis; and ITT analysis is used.
- *Fair*: One or more of the following issues is found in the study: some though not major differences between groups exist at follow-up; measurement instruments are acceptable but not ideal, though are generally applied equally; some but not all important outcomes are considered; some but not all potential confounders are accounted for in analyses. ITT analysis must be done.
- *Poor*: One or more of the following “fatal flaws” is found in the study: initially assembled groups are not comparable or maintained throughout the study; unreliable or invalid measurements are used or applied unequally across groups; key confounders are given little to no attention in analyses; ITT analysis is not used.

Critical appraisal assessments were used for sensitivity analyses by excluding poor quality studies to evaluate the robustness of findings.

### Data synthesis

The primary aim of this systematic review was to determine effects of SJW on depressive symptoms, quality of life, and adverse events compared with placebo and active comparators. We differentiated effectiveness and comparative effectiveness analyses. Placebo trials were used to estimate the treatment effect of SJW by demonstrating effects that go beyond placebo effects. A further key aim of the review was to determine the comparative effectiveness of SJW compared with standard antidepressant treatment (both psychotherapy or antidepressant medication). Comparative effectiveness results and equivalence assessments of the efficacy and safety took the consistency of effects across individual studies and the statistical power to detect a statistically significant difference between treatment groups into account. For all efficacy outcomes and the number of patients with adverse events, we used the Hartung-Knapp-Sidik-Jonkman method for a random effects meta-analysis [12–14]. For specific adverse events, many of which are very rare, we used exact conditional methods to estimate ORs and CIs. Heterogeneity was assessed using the I^2^ statistic and values above 75 % were interpreted as possibly representing considerable heterogeneity.

We conducted preplanned subgroup analyses for different patient groups depending on the severity of depression. In studies comparing SJW to antidepressant medication we differentiated selective serotonin reuptake inhibitors (SSRIs), tricyclic antidepressants (imipramine, amitriptyline), and other (e.g., maprotiline, Deanxit). Further meta-regressions were conducted to identify sources of heterogeneity across studies where appropriate. We conducted sensitivity analyses to test the robustness of results (e.g., to test effects in studies with sufficient power to detect effect differences between study arms or excluding poor quality studies). Publication bias was assessed with the Begg and Egger tests; in the case of indications for bias, treatment estimates were estimated using the trim-and-fill method.

### Quality of evidence

The quality of evidence was assessed using the GRADE approach [15]. The body of evidence was evaluated on the following dimensions: *study limitations*, *inconsistency*, *directness*, and *precision*. The quality was downgraded when results were primarily based on studies with substantial limitations and suspected risk of bias; when results were inconsistent across individual studies or the result was based on a single study without replication in an independent research study; in the presence of substantial heterogeneity in pooled analyses and variation in the direction of effects; when conclusions were based on indirect evidence (e.g., effects bases on subgroup analyses or meta-regressions in the absence of head-to-head comparisons); and when pooled results were imprecise estimates of the treatment effect with wide confidence intervals spanning effect sizes with different clinical conclusions. The quality of evidence was graded on a 4-item scale:

- *High* indicates that review authors are very confident that the effect estimate lies close to the true effect for a given outcome, as the body of evidence has few or no deficiencies. As such, the reviewers believe the findings are stable and further research is very unlikely to change confidence in the effect estimate.
- *Moderate* indicates that the review authors are moderately confident that the effect estimate lies close to the true effect for a given outcome, as the body of evidence has some deficiencies. As such, the reviewers believe that the findings are likely to be stable, but further research may change confidence in the effect estimate and may even change the estimate.
- *Low* indicates that the review authors have limited confidence that the effect estimate lies close to the true effect for a given outcome, as the body of evidence has major or numerous (or both) deficiencies. As such, the reviewers believe that additional evidence is needed before concluding either that the findings are stable or that the effect estimate lies close to the true effect.
- *Very low* indicates that the review authors have very little confidence that the effect estimate lies close to the true effect for a given outcome, as the body of evidence has very major deficiencies. As such, the true effect is likely to be substantially different from the estimated effect; thus, any estimate of effect is very uncertain.

This review was registered in PROSPERO CRD42015016406.

## Results

**Fig. 1:**
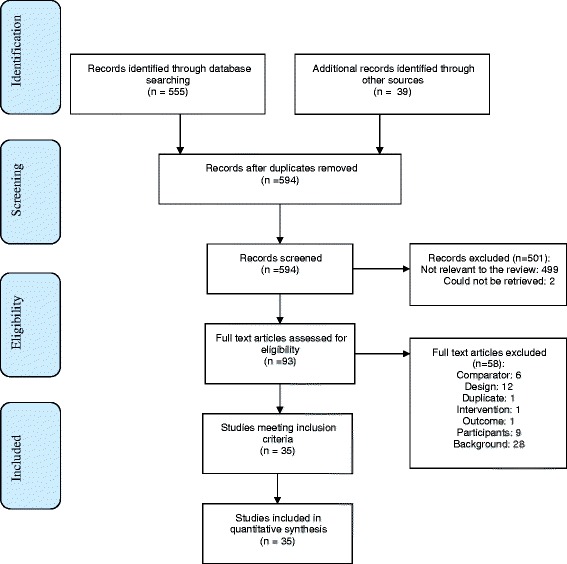
Article flow diagram

We identified 594 potentially relevant citations through the electronic database search and reference mining. We obtained 93 studies as full text. In total, 35 studies met inclusion criteria (see Fig. 1 for PRISMA diagram) [16–50]. All studies addressed the efficacy of SJW reporting on the rate of treatment responders, mean scores on depression scales, or the number of patients in remission. Very few studies reported on relapse and quality of life and studies. In total, 34 studies addressed safety and reported on the number of patients with adverse events or the frequency of individual events. Risk of bias in included studies varied: ten studies were rated “good,” 14 “fair,” and 11 “poor” quality (see Table 1). Table 2 shows key characteristics of the included studies.

**Table 1:** Study quality/risk of bias for individual included studies

Study ID	Recruitment method (random sequence generation)	Allocation concealment	Blinding of participants and personnel	Blinding of outcome assessment	Incomplete outcome data	Selective reporting of outcome data	Other: all receive TAU, only treatment group receives SJW (no placebo for controls)	Other: appropriate washout period or exclusion of individuals taking personal supplements	Other: baseline assessment, appropriate statistical analysis, COI)	USPSTF quality rating (good, fair, poor)
Behnke, 2002 [17]	Unclear risk	Unclear risk	Unclear risk	Unclear risk	Low risk	Unclear risk	Low risk	NA	Low risk	Poor
Bernhardt, 1993 [16]	Unclear risk	Unclear risk	High risk	High risk	Unclear risk	Unclear risk	Low risk	NA	Unclear risk	Poor
Bjerkenstedt, 2005 [18]	Unclear risk	Unclear risk	Low risk	Low risk	Low risk	Unclear risk	Low risk	NA	Low risk	Fair
Brenner, 2000 [19]	Unclear risk	Unclear risk	Low risk	Unclear risk	Low risk	Unclear risk	Low risk	NA	Low risk	Fair
Fava, 2005 [20]	Unclear risk	Unclear risk	Low risk	Unclear risk	Low risk	Unclear risk	Low risk	NA	Low risk	Poor
Gastpar, 2005 [21]	Low risk	Unclear risk	Low risk	Unclear risk	Unclear risk	Unclear risk	Low risk	NA	Low risk	Poor
Gastpar, 2006 [22]	Low risk	Unclear risk	Low risk	Unclear risk	Low risk	Unclear risk	Low risk	NA	Low risk	Good
HDTSG, 2002 [23]	Low risk	Low risk	Low risk	Low risk	Unclear risk	Low risk	Low risk	NA	Low risk	Fair
Hangsen, 1994 [48]	Low risk	Low risk	Low risk	Unclear risk	Unclear risk	Unclear risk	Low risk	NA	Low risk	Poor
Harrer, 1993 [24]	Low risk	Unclear risk	Low risk	Unclear risk	Unclear risk	Unclear risk	Low risk	NA	Low risk	Poor
Harrer, 1999 [25]	Unclear risk	Unclear risk	Unclear risk	Unclear risk	Low risk	Unclear risk	Low risk	NA	Low risk	Fair
Kalb, 2001 [26]	Low risk	Unclear risk	Low risk	Unclear risk	Low risk	Unclear risk	Low risk	NA	Low risk	Good
Kasper, 2006 [27]	Low risk	Low risk	Low risk	Low risk	Unclear risk	Low risk	Low risk	NA	Low risk	Fair
Kasper, 2008 [28]	Low risk	Low risk	Low risk	Unclear risk	Low risk	Low risk	Low risk	Low risk	Low risk	Fair
Laakmann, 1998 [29]	Low risk	Unclear risk	Low risk	Unclear risk	Low risk	Unclear risk	Low risk	NA	Low risk	Good
Lecrubier, 2002 [30]	Unclear risk	Unclear risk	Low risk	Unclear risk	Low risk	Unclear risk	Low risk	NA	Low risk	Fair
Lenoir, 1999 [31]	Unclear risk	Low risk	Unclear risk	Unclear risk	Unclear risk	Unclear risk	Low risk	NA	Low risk	Poor
Liu, 2010 [32]	High risk	Unclear risk	High risk	Unclear risk	Low risk	Unclear risk	Low risk	NA	Low risk	Poor
Mannel, 2010 [33]	Low risk	Low risk	Low risk	Unclear risk	Low risk	Unclear risk	Low risk	NA	Low risk	Good
Montgomery, 2000 [34]	Unclear risk	Unclear risk	Unclear risk	Unclear risk	Low risk	Unclear risk	Low risk	NA	Unclear risk	Poor
Moreno, 2005 [35]	Unclear risk	Unclear risk	Unclear risk	Unclear risk	Low risk	Unclear risk	Low risk	NA	Low risk	Fair
Pakseresht, 2012 [36]	Unclear risk	Unclear risk	Unclear risk	Unclear risk	Low risk	Unclear risk	Low risk	NA	Low risk	Fair
Philipp, 1999 [37]	Unclear risk	Unclear risk	Low risk	Unclear risk	Low risk	Unclear risk	Low risk	NA	Low risk	Fair
Rahman, 2008 [38]	Low risk	Low risk	Low risk	Unclear risk	Unclear risk	Unclear risk	Low risk	NA	Low risk	Poor
Schrader, 1998 [40]	Low risk	Unclear risk	Low risk	Unclear risk	Low risk	Unclear risk	Low risk	NA	Low risk	Good
Schrader, 2000 [39]	Unclear risk	Unclear risk	Low risk	Unclear risk	Low risk	Unclear risk	Low risk	NA	Low risk	Fair
Shelton, 2001 [41]	Low risk	Low risk	Low risk	Unclear risk	Low risk	Unclear risk	Low risk	NA	Low risk	Good
Szegedi, 2005 [42]	Low risk	Low risk	Low risk	Unclear risk	Low risk	Unclear risk	Low risk	NA	Low risk	Fair
Uebelhack, 2004 [43]	Low risk	Unclear risk	Low risk	Unclear risk	Low risk	Unclear risk	Low risk	NA	Low risk	Good
Volz, 2000 [50]	Unclear risk	Low risk	Low risk	Low risk	Low risk	Unclear risk	Low risk	NA	Low risk	Good
Vorbach, 1997 [44]	Low risk	Unclear risk	Low risk	Unclear risk	Unclear risk	Unclear risk	Low risk	NA	Low risk	Poor
Wheatley, 1997 [45]	Low risk	Unclear risk	Low risk	Unclear risk	Low risk	Unclear risk	Low risk	NA	Low risk	Good
Witte. 1995 [49]	Unclear risk	Low risk	Low risk	High risk	Low risk	Unclear risk	Low risk	NA	Low risk	Good
Woelk, 2000 [46]	Low risk	Unclear risk	Low risk	Unclear risk	Low risk	Unclear risk	Low risk	NA	Low risk	Fair
van Gurp, 2002 [47]	Low risk	Low risk	Low risk	Low risk	Low risk	Unclear risk	Low risk	NA	Low risk	Fair

*SJW* St. John’s wort, *ITT* intention-to-treat analysis, *TAU* treatment as usual

**Table 2:** Evidence table

Study Details	Participants	Intervention
Behnke, 2002 [17], Country: NR Funding: unclear, industry author, provided SJW	Number of participants: 70 Diagnosis: clinical rating scale for depression, MDD (ICD) Severity: mild-moderate Age (years, M (SD)): 18–73 overall; 51.4 (10.9) SJW; 48.0 (12.6) fluoxetine, gender (% male): 29 % SJW; 34 % fluoxetine; (1 participant missing from SJW group)	Extract: Hypericum perforatum Dosage: 150 mg (0.450–0.495 mg total hypericin), 6 weeks Comparator: fluoxetine Follow-up time: 6 weeks
Bernhardt, 1993 [16], Country: Germany Funding: unclear, not reported	Number of participants: 55 Diagnosis: clinical rating scale for depression Severity: mild-moderate Age (years, M (SD)): 54.5 (11.6), gender (% male): 29 %	Extract: hypericin Dosage: 0.25 mg extract/3 times per day (morning/noon/night), 4 weeks; Comparator: 0.25 mg extract/3 times per day (2 in the morning, 1 at noon) Follow-up time: 4 weeks
Bjerkenstedt, 2005 [18], Country: Sweden Funding: no industry funding	Number of participants: 174 Diagnosis: MDD (DSM), clinical rating scale for depression Severity: mild-moderate Age (years, M (SD)): 49.1 (12.0) SJW; 50.4 (11.6) fluoxetine; 51.4 (11.8) placebo; gender (% male): SJW 20 %; fluoxetine 24 %: placebo 18 %	Extract: hypericum LI 160 Dosage: 300 mg, 3 times per day, daily, 4 weeks Comparator: fluoxetine, placebo Follow-up time: 4 weeks
Brenner, 2000 [19], Country: USA Funding: no industry funding	Number of participants: 30 Diagnosis: MDD (DSM) and clinical rating scale for depression Severity: mild-moderate Age (years, M (SD)): 45; gender (% male): 37 %	Extract: LI 160 Dosage: 600 mg per day during week 1, followed by 900 mg per day for the remainder of the trial Comparator: sertraline Follow-up time: 7 weeks
Fava, 2005 [20], Country: USA Funding: no industry funding	Number of participants: 135 Diagnosis: MDD (DSM), clinical rating scale for depression, other diagnosis, SCID Severity: moderate Age (years, M (SD)): 37.3 (11.0) Gender (% male): 43 %	Extract: LI 160 Dosage: 300 mg, 3 times a day Comparator: fluoxetine, placebo Follow-up time: 12 weeks
Gastpar, 2005 [21] Country: Germany Funding: unclear, industry author, provided SJW	Number of participants: 241 Diagnosis: MDD (DSM), clinical rating scale for depression, MDD (ICD) Severity: moderate Age (years, M (SD)): 48.3 (12.7) SJW; 49.5 (13.8) sertraline Gender (% male): SJW 21 %; sertraline 31 %	Extract: STW3 Dosage: 612 mg/day/12 weeks Comparator: sertraline Follow-up time: 24 weeks
Gastpar, 2006 [22], Country: Germany Funding: unclear, industry author, provided SJW	Number of participants: 388 Diagnosis: MDD (DSM), clinical rating scale for depression, MDD (ICD) Severity: moderate Age (years, M (SD)): SJW 50.8 (12.1); citalopram 49.3 (10.7); placebo 49.4 (12.7) Gender (% male): SJW 34 %, citalopram 35 %, placebo 27 %	Extract: STW3-VI Dosage: 900 mg/day/6 weeks Comparator: citalopram, placebo Follow-up time: 6 weeks
HDTSG, 2002 [23], Country: USA Funding: no industry funding	Number of participants: 338 Diagnosis: MDD (DSM), clinical rating scale for depression Severity: not reported Age (years, M (SD)): SJW 43.1 (13.5); sertraline 43.9 (13.9); placebo 40.1 (12.2) Gender (% male): SJW 35 %; sertraline 33 %; placebo 34 %	Extract: LI 160 Dosage: 300 mg/3 times a day/8 weeks Comparator: sertraline, placebo Follow-up time: 8 weeks
Hangsen, 1994 [48], Country: Germany Funding: unclear, industry author, provided SJW	Number of participants: 108 Diagnosis: MDD (DSM), clinical rating scale for depression Severity: not reported Age (years, M (SD)): 53.0 (7.5) SJW; 53.5 (10.3) placebo Gender (% male): SJW 42 %; placebo 32 %	Extract: LI 160 Dosage: 300 mg/3 times a day/6 weeks Comparator: placebo Follow-up time: 5 and 6 weeks
Harrer, 1994 [24], Country: Germany Funding: unclear, not reported	Number of participants: 102 Diagnosis: clinical rating scale for depression, MDD (ICD) Severity: moderate Age (years, M (SD)): SJW 43.8 (13.4); maprotiline 47.6 (10.9) Gender (% male): SJW 25 %; maprotiline 31 %	Extract: LI 160 Dosage: 300 mg/3 times a day/4 weeks Comparator: maprotiline Follow-up time: 4 weeks
Harrer, 1999 [25], Country: NR Funding: unclear, not reported	Number of participants: 228 Diagnosis: MDD (ICD) Severity: mild-moderate Age (years, M (SD)): SJW 68.4; fluoxetine 69.1 Gender (% male): 13 %	Extract: LoHyp-57 Dosage: 400 mg/two times a day/6 weeks Comparator: fluoxetine Follow-up time: 6 weeks
Kalb, 2001 [26], Country: Germany Funding: industry funding	Number of participants: 72 Diagnosis: MDD (DSM), clinical rating scale for depression Severity: mild-moderate Age (years, M (SD)): SJW 48 (11); placebo 49 (10) Gender (% male): SJW 30 %; placebo 37 %	Extract: WS 5572 Dosage: 300 mg/3 times a day/6 weeks Comparator: placebo Follow-up time: 6 weeks
Kasper, 2006 [27], Country: Germany Funding: unclear, industry author, provided SJW	Number of participants: 332 Diagnosis: MDD (DSM), clinical rating scale for depression Severity: mild-moderate Age (years, M (SD)): SJW 600 mg 46.3 (11.5); SJW 1200 mg 46.1 (10.7); placebo 46.9 (11.8) Gender (% male): SJW 600 mg 44 %; SJW 1200 mg 34 %; placebo 31 %	Extract: WS 5570 Dosage: 600 or 1200 mg/day/6 weeks Comparator: placebo Follow-up time: 6 weeks
Kasper, 2008 [28], Countries: Germany and Sweden Funding: industry funding	Number of participants: 570 Diagnosis: MDD (DSM), clinical rating scale for depression, MDD (ICD) Severity: mild Age (years, M (SD)): 47.5 (10.7); placebo 47.4 (11.8) Gender (% male): SJW 27 %; placebo 24 %	Extract: WS 5570 Dosage: 300 mg/3 times a day/26 weeks Comparator: placebo Follow-up time: 32 weeks
Laakmann, 1998 [29], Country: Germany Funding: unclear, industry author, provided SJW	Number of participants: 196 Diagnosis: MDD (DSM), clinical rating scale for depression Severity: mild-moderate Age (years, M (SD)): SJW WS 5572 47.3 (11.8); SJW WS 5573 48.7 (11.8); placebo 51.0 (12.7) Gender (% male): SJW WS 5572 18 %; SJW WS 5573 14 %; placebo 29 %	Extract: WS 5572; WS 5573 Dosage: 3 × 300 mg/day/6 weeks Comparator: placebo Follow-up time: 6 weeks
Lecrubier, 2002 [30], Country: France Funding: industry funding	Number of participants: 375 Diagnosis: MDD (DSM), clinical rating scale for depression Severity: mild-moderate Age (years, M (SD)): SJW 40.2 (11.7); placebo 41.2 (11.4) Gender (% male): SJW 24 %; placebo 23 %	Extract: WS 5570 Dosage: 300 mg/3 times a day/6 weeks Comparator: placebo Follow-up time: 6 weeks
Lenoir, 1999 [31], Countries: Switzerland and Germany Funding: unclear, industry author, provided SJW	Number of participants: 348 Diagnosis: MDD (ICD) Severity: mild-moderate Age (years, M (SD)): 19–94 (range) Gender (% male): 26 %	Extract: hypericin Dosage: 0.17 mg, 0.33 mg, or 1 mg/day (divided into 3 doses)/6 weeks Comparator: other doses of SJW
Liu, 2010 [32], Country: China Funding: no industry funding	Number of participants: 170 Diagnosis: clinical rating scale for depression, other diagnosis, ISFC and WHO criteria Severity: not reported Age (years, M (SD)): SJW 67 (2.7); Deanxit 68 (2.8); psychotherapy 68 (3.0); placebo 67 (2.5) Gender (% male): 50 %	Extract: NA Dosage: 300 mg/3 times a day/12 weeks Comparator: Deanxit 10.5 mg/daily, cognitive therapy, suggestion therapy, supportive therapy and rational emotive therapy/twice per week, placebo (oryzanol 20 mg)/3 times a day Follow-up time: 12 weeks
Mannel, 2010 [33], Country: Germany Funding: industry funding	Number of participants: 201 Diagnosis: clinical rating scale for depression, MDD (ICD) Severity: mild-moderate Age (years, M (SD)): SJW 47.0 (13.1); placebo 46.6 (13.8) Gender (% male): 17 %	Extract: LI 160 Dosage: 300 g/2 times a day/8 weeks Comparator: placebo Follow-up time: 8 weeks
Montgomery, 2000 [34], Country: UK Funding: unclear, not reported	Number of participants: 248 Diagnosis: MDD (DSM) Severity: mild-moderate Age (years, M (SD)): NA Gender (% male): NA	Extract: LI 160 Dosage: 300 mg/3 times a day/12 weeks Comparator: placebo Follow-up time: 6 weeks
Moreno, 2005 [35], Country: Brazil Funding: industry funding	Number of participants: 66 Diagnosis: clinical rating scale for depression Severity: mild-moderate Age (years, M (SD)): 40.5 (10.7) Gender (% male): 17 %	Extract: NA Dosage: 300 mg/3 times a day/8 weeks Comparator: fluoxetine, placebo Follow-up time: 8 weeks
Pakseresht, 2012 [36], Country: Iran Funding: no industry funding	Number of participants: 40 Diagnosis: clinical rating scale for depression, other diagnosis, diagnosed depression, method unspecified Severity: mild-moderate Age (years, M (SD)): SJW 29.8 (6.2); placebo 30 (16.6) Gender (% male): SJW 50 %; placebo 45 %	Extract: NA Dosage: 300 mcg/3 times a day/6 weeks Comparator: nortriptyline 75–100 mg, imipramine 100–150 mg, amitriptyline 100–150 mg/daily, placebo/daily Follow-up time: 6 weeks
Philipp, 1999 [37], Country: Germany Funding: industry funding	Number of participants: 263 Diagnosis: clinical rating scale for depression, MDD (ICD) Severity: not reported Age (years, M (SD)): 47 (12) Gender (% male): 25 %	Extract: STEI 300 Dosage: 350 mg/3 times day/8 weeks Comparator: imipramine, placebo Follow-up time: 8 weeks
Rahman, 2008 [38], Country: Pakistan Funding: industry funding	Number of participants: 225 Diagnosis: clinical rating scale for depression, MDD (ICD) Severity: mild-moderate Age (years, M (SD)): SJW 33.89 (10.884); placebo 36.29 (12.478) Gender (% male): SJW 23 %; placebo 21 %	Extract: NA Dosage: 300 mg/3 times a day/6 weeks Comparator: placebo Follow-up time: 6 weeks
Schrader, 1998 [40] Country: Germany Funding: unclear, industry author, provided SJW	Number of participants: 162 Diagnosis: clinical rating scale for depression, MDD (ICD) Severity: mild-moderate Age (years, M (SD)): SJW 47 (32–59.25, 25–75 % range); placebo 39 (30–59.25, 25–75 % range) Gender (% male): SJW 28 %; placebo 38 %	Extract: ZE117 Dosage: 250 mg/2 times a day/6 weeks Comparator: placebo Follow-up time: 6 weeks
Schrader, 2000 [39] Country: Germany Funding: industry funding	Number of participants: 240 Diagnosis: clinical rating scale for depression, MDD (ICD) Severity: mild-moderate Age (years, M (SD)): SJW 46 (19); fluoxetine 47 (17) Gender (% male): SJW 29 %; fluoxetine 41 %	Extract: Ze 117 Dosage: 250 mg/2 times a day/6 weeks Comparator: fluoxetine Follow-up time: 6 weeks
Shelton, 2001 [41], Country: USA Funding: unrestricted grant/industry funding but no conflict	Number of participants: 200 Diagnosis: MDD (DSM), clinical rating scale for depression Severity: Mild-moderate Age (years, M (SD)): SJW 41.4 (12.5); placebo 43.3 (13.7) Gender (% male): SJW 35 %; placebo 37 %	Extract: NA Dosage: 300 mg/a day/8 weeks Comparator: placebo Follow-up time: 8 weeks
Szegedi, 2005 [42], Country: Germany Funding: industry funding	Number of participants: 251 Diagnosis: MDD (DSM), clinical rating scale for depression Severity: moderate-severe Age (years, M (SD)): SJW 49.0 (11.0); Paroxetine 45.5 (11.5) Gender (% male): SJW 30 %; paroxetine 32 %	Extract: WS 5570 Dosage: 300 mg–600 mg/3 time a day/6 weeks Comparator: paroxetine Follow-up time: 6 weeks
Uebelhack, 2004 [43], Country: Germany Funding: unclear, not reported	Number of participants: 140 Diagnosis: MDD (DSM), clinical rating scale for depression, MDD (ICD) Severity: not reported Age (years, M (SD)): SJW 46.4 (12.5); placebo 43.3 (12.6) Gender (% male): SJW 30 %; placebo 36 %	Extract: STW 3-VI Dosage: 900 mg/day/6 weeks Comparator: placebo Follow-up time: 6 weeks
Volz, 2000 [50], Country: Germany Funding: unclear, industry author, provided SJW	Number of participants: 140 Diagnosis: MDD (DSM), clinical rating scale for depression Severity: mild-moderate Age (years, M (SD)): 47 Gender (% male): 19 %	Extract: D-0496 (hypericin) Dosage: 250 mg/twice a day/6 weeks Comparator: placebo Follow-up time: 6–8 weeks
Vorbach, 1997 [44], Country: Germany Funding: unclear, industry author, provided SJW	Number of participants: 209 Diagnosis: MDD (ICD) Severity: not reported Age (years, M (SD)): SJW 48.8 (12.0); imipramine 50.1 (11.8) Gender (% male): SJW 27 %; imipramine 25 %	Extract: LI 160 Dosage: 3 × 600 mg/day/6 weeks Comparator: imipramine Follow-up time: 6 weeks
Wheatley, 1997 [45], Country: UK Funding: unclear, not reported	Number of participants: 165 Diagnosis: MDD (DSM), clinical rating scale for depression Severity: Mild-moderate Age (years, M (SD)): SJW 42 (range: 20–64); Amitriptyline 38 (range: 24–65) Gender (% male): SJW 16 %; Amitriptyline 23 %	Extract: LI 160 Dosage: 300 mg/3 times a day/6 weeks Comparator: Amitriptyline Follow-up time: 6 weeks
Witte, 1995 [49], Country: Germany Funding: unclear, not reported	Number of participants: 97 Diagnosis: clinical rating scale for depression, MDD (ICD) Severity: Not reported Age (years, M (SD)): 44.7 (10.9) SJW; 41.6 (12.5) placebo Gender (% male): SJW 31 %; placebo 37 %	Extract: psychotonin forte Dosage: 100–120 mg/2 times a day/6 weeks Comparator: placebo Follow-up time: 6 weeks
Woelk, 2000 [46], Country: Germany Funding: unrestricted grant/industry funding but no conflict	Number of participants: 324 Diagnosis: clinical rating scale for depression, MDD (ICD) Severity: mild-moderate Age (years, M (SD)): SJW 46.5 (12.7); imipramine 45.4 (12.8) Gender (% male): SJW 29 %; imipramine 29 %	Extract: Ze 117 Dosage: 250 mg/2 times a day/6 weeks Comparator: imipramine Follow-up time: 6 weeks
van Gurp, 2002 [47], Country: Canada Funding: unrestricted grant/industry funding but no conflict	Number of participants: 90 Diagnosis: MDD (DSM), clinical rating scale for depression Severity: not reported Age (years, M (SD)): SJW 40.9 (11.6); sertraline 39.1 (10.2) Gender (% male): SJW 36 %; sertraline 42 %	Extract: NR Dosage: 1–2 300 mg/3 times a day/12 weeks Comparator: sertraline Follow-up time: 12 weeks

*NA* not applicable, *NR* not reported

The summary of findings table (Table 3) summarizes the review findings by comparator and outcome, the GRADE score, and the reason for downgrading the quality of evidence, where applicable.

**Table 3:** Summary of findings and quality of evidence table

Outcome	Study design: number of studies, number of participants	Findings: direction and magnitude of effect	GRADE
Comparison: SJW vs. placebo			
Depression, number of treatment responders	18 RCTs, *N* = 2922	RR 1.53 (1.19, 1.97), favors SJW	Moderate^a^
Depression scale score	16 RCTs, *N* = 2888	SMD 0.49 (0.23, 0.74), favors SJW	Moderate^a^
Depression remission	9 RCTs, *N* = 1419	RR 1.69 (0.63, 4.55), n.s.	Low^a,b^
Depression relapse	1 RCT, *N* = 426	RR 0.70 (0.49, 1.02), n.s.	Very low^a,b^
Quality of life—mental	2 RCTs, *N* = 358	SMD 0.48 (0.24, 0.73), favors SJW	Low^b^
Quality of life—physical	2 RCTs, *N* = 358	SMD 0.28 (−1.03, 0.47), n.s.	Very low^a,b^
Number of patients with adverse events	13 RCTs, *N* = 2600	OR 0.83 (0.62, 1.13), n.s.	Moderate^b^
Serious adverse events	6 RCTs, *N* = 1358	OR 0.26 (0.04, 1.23), n.s.	Moderate^a^
Gastrointestinal/metabolic/nutritional adverse events	16 RCTs, *N* = 2490	OR 1.06 (0.83, 1.41), n.s.	Low^a,b^
Neurologic/nervous system adverse events	14 RCTs, *N* = 2404	OR 1.56 (1.08, 3.32) SJW more AE	Low^a,b^
Skin/musculoskeletal adverse events	10 RCTs, *N* = 1978	OR 1.47 (0.98, 2.21), n.s.	Very low^a,b^
Photosensitivity	4 RCTs, *N* = 1054	OR 1.10 (0.36, 3.56), n.s.	Low^a,b^
Respiratory/infectious adverse events	7 RCTs, *N* = 1081	OR 1.48 (0.95, 2.33), n.s.	Low^a,b^
Other organ system (eye, ear, liver, renal, reproductive) adverse events	5 RCTs, *N* = 1054	OR 1.87 (1.08, 3.32), SJW more AE	Low^a,b^
Cardiovascular adverse events	4 RCTs, *N* = 759	OR 6.81 (0.92, 304.08), n.s.	Very low^a,b,d^
Psychiatric adverse events	3 RCTs, *N* = 608	OR 1.61 (0.34, 10.21), n.s.	Very low^a,b,d^
Sexual dysfunction adverse events	2 RCTs, *N* = 428	OR 1.92 (0.94, 4.00), n.s.	Very low^a,b,d^
Comparison: SJW vs. antidepressant			
Depression, number of treatment responders	17 RCTs, *N* = 2776	RR 1.01 (0.90, 1.14), n.s.	Moderate^b^
Depression scale score	14 RCTs, *N* = 2248	SMD 0.03 (−0.15, 0.21), n.s.	Moderate^b^
Depression remission	7 RCTs, *N* = 787	RR 1.17 (0.84, 1.62), n.s.	Low^b^
Depression relapse	1 RCT, *N* = 241	RR 4.17 (0.47, 33.33), n.s.	Very low^a,b^
Quality of life—mental	1 RCT, *N* = 216	SMD −0.11 (−0.15, 0.38), n.s.	Very low^a,b^
Quality of life—physical	1 RCT, *N* = 153	SMD 0.35 (0.01, 0.70), favors SJW	Very low^a,b^
Number of patients with adverse events	11 RCTs, *N* = 1946	OR 0.67 (0.56, 0.81), favors SJW	Moderate^a^
Serious adverse events	4 RCTs, *N* = 805	OR 0.62 (0.05, 5.46) n.s.	Low^a,b^
Gastrointestinal/metabolic/nutritional adverse events	15 RCTs, *N* = 2491	OR 0.43 (0.34, 0.55) favors SJW	Low^a,b^
Neurologic/nervous system adverse events	15 RCTs, *N* = 2492	OR 0.29 (0.24, 0.36) favors SJW	Low^a,b^
Skin/musculoskeletal adverse events	10 RCTs, *N* = 1587	OR 1.18 (0.79, 1.78) n.s.	Low^a,b^
Respiratory/infectious adverse events	2 RCTs, *N* = 352	OR 1.25 (0.70, 2.25) n.s.	Very low^a,b^
Other organ system (eye, ear, liver, renal, reproductive) adverse events	4 RCTs, *N* = 761	OR 0.85 (0.52, 1.38), n.s.	Low^a,b^
Cardiovascular adverse events	5 RCTs, *N* = 750	OR 0.55 (0.26, 1.16), n.s.	Low^a,b^
Psychiatric adverse events	4 RCTs, *N* = 552	OR 0.41 (0.19, 0.87) favors SJW	Very low^a,b^
Sexual dysfunction adverse events	2 RCTs, *N* = 301	OR 0.51 (0.30, 0.88) favors SJW	Low^a,b^
Effect of depression severity			
Depression, treatment responders	18 RCTs, *N* = 2922	Meta-regression did not suggest differences between patient subgroups (*p* = 0.798)	Very low^a,c^
Depression scale score	16 RCTs, *N* = 2888	Meta-regression did not suggest differences between patient subgroups (*p* = 0.365)	Very low^a,c^
Depression remission	9 RCTs, *N* = 1507	Meta-regression did not suggest differences between patient subgroups (*p* = 0.159)	Very low^a,c^
Number of patients with adverse events	13 RCTs, *N* = 2600	Meta-regression did not suggest differences between patient subgroups (*p* = 0.480)	Very low^a,c^

Quality of evidence was downgraded (by 1 or by 2, depending on the severity) for the following

^a^Inconsistency (heterogeneity, direction of effects; no replication)

^b^Study limitations (no good quality study; effect not present when excluding poor quality studies; studies not designed or not powered to assess outcome)

^c^Indirectness (no head-to-head trials, effect based on indirect comparison)

^d^Imprecision (wide confidence interval)

*SJW* St. John’s wort, *n.s.* not statistically significantly different, *AE* adverse event

*Review question 1: What are the efficacy and safety of SJW in adults with MDD compared to placebo or active comparator?*

To answer our first research question, we examined the efficacy and safety of SJW compared to both placebo and standard antidepressant treatment.

### SJW vs. placebo

**Fig. 2:**
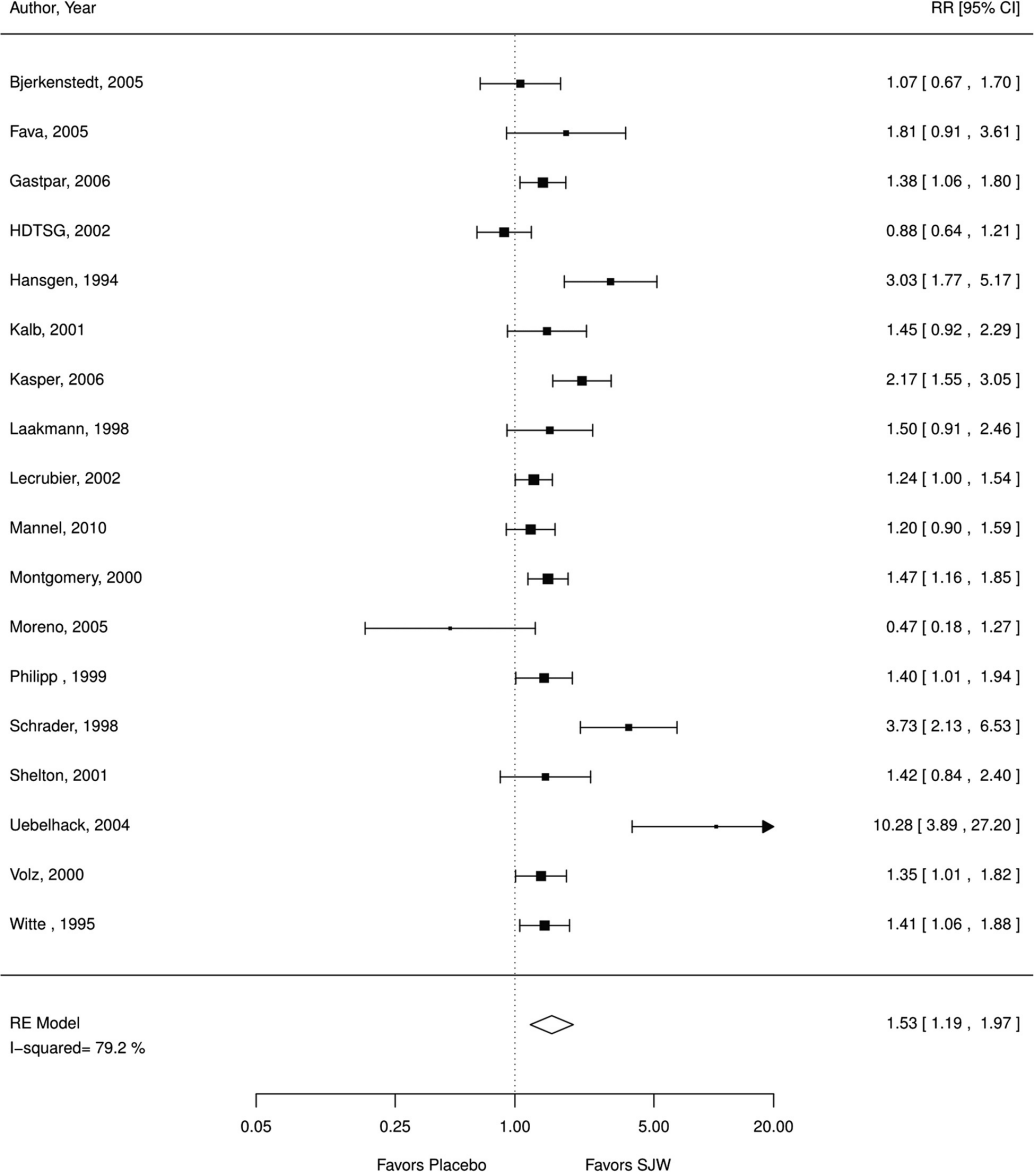
SJW vs. placebo, treatment responder rate; *RE* random effects, *RR* relative risk, *SJW* St. John’s wort

**Fig. 3:**
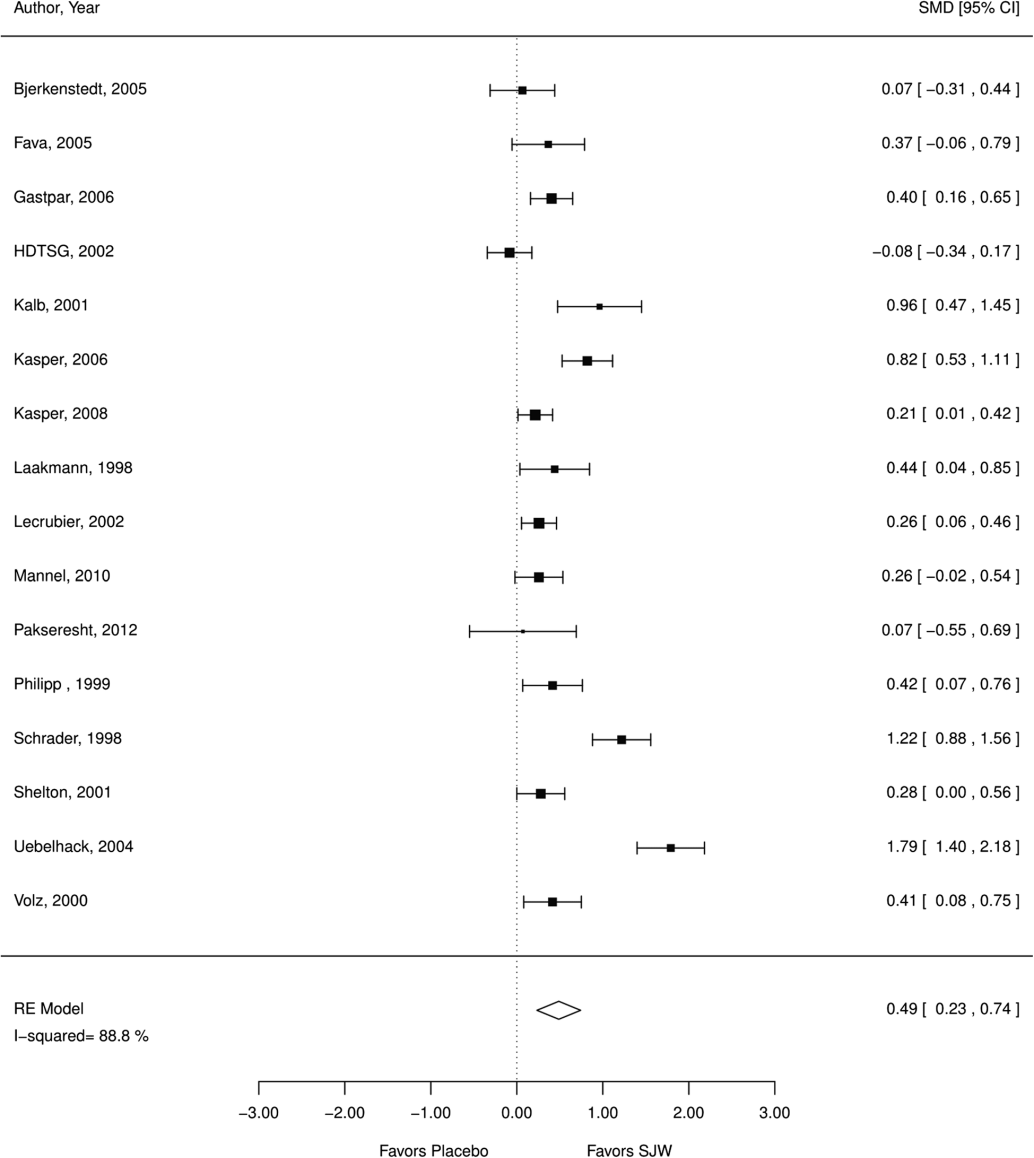
SJW vs. placebo, depression scale standardized mean differences; *RE* random effects, *SJW* St. John’s wort, *SMD* standardized mean differences

*a. Efficacy*. We found evidence that SJW is associated with statistically significant improvement in depression symptoms compared to placebo. SJW groups reported significantly more treatment responders (RR 1.53; CI 1.19, 1.97; I^2^ 79 %; 18 RCTs; *N* = 2922; Fig. 2). Participants receiving SJW also had significantly lower mean depression scale scores (SMD 0.49; CI 0.23, 0.74; 16 RCTs; I^2^ 89 %, *N* = 2888; Fig. 3) than participants receiving a placebo. Both analyses indicated substantial heterogeneity that lowered the quality of evidence. Sensitivity analyses showed very similar results when excluding poor quality studies indicating that the effects of SJW were not primarily driven by poor methodological quality.

**Fig. 4:**
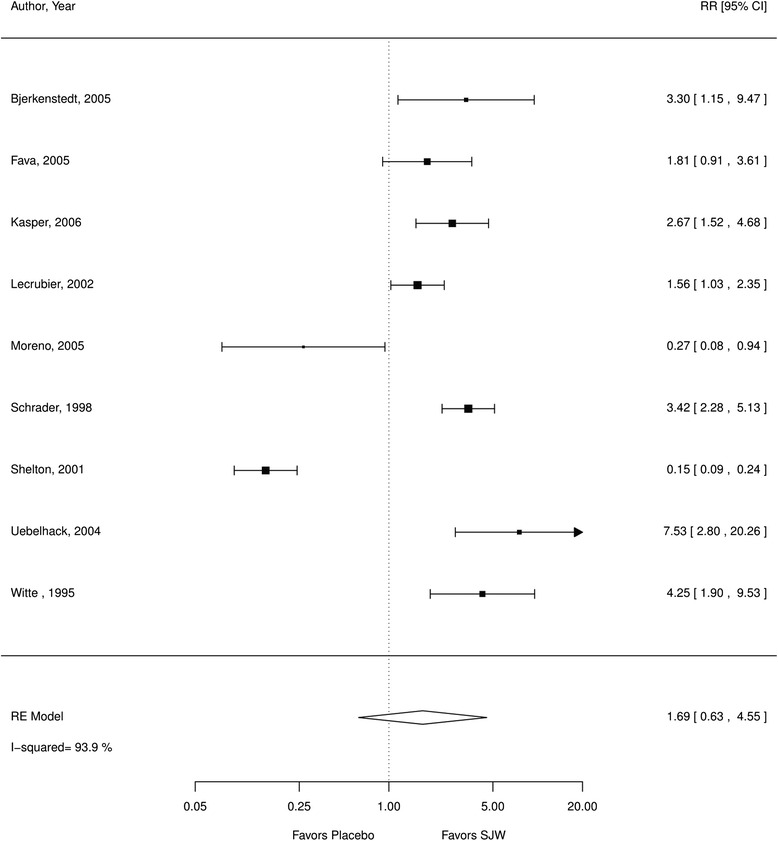
SJW vs. placebo, number of patients in remission; *RE* random effects, *RR* relative risk, *SJW* St. John’s wort

We found no statistically significant difference in the number of patients in remission comparing SJW and placebo (RR 1.69; CI 0.63 to 4.55; 9 RCTs; I^2^ 94 %, *N* = 1419; Fig. 4). However, there was considerable heterogeneity which lowered the quality of evidence assessment and the direction of effects varied across studies: in the majority favoring SJW but two studies reported more patients in remission in the placebo arm. Results were similar when excluding poor quality studies and between-study heterogeneity was not reduced. In the majority of studies the number of patients in remission was small in both treatment arms. The median follow-up time across studies was 6 weeks (range 4–12 weeks).

Relapse was only assessed in one study without replication by another study and did not indicate a statistically significant difference between SJW and placebo. Quality of life was assessed in two fair quality trials; SJW treatment effects were shown to be superior for the mental but not for the physical component (see Table 3).

*b. Safety.* Most (34/35) of the included studies addressed the safety of SJW, but rigor of assessment varied greatly. In the included RCTs, SJW was not more likely to cause patients to experience adverse events than placebo overall (OR 0.83; CI 0.62, 1.13; 13 RCTs, Table 3). The total number of serious adverse events also did not differ significantly between patients who were administered SJW and those who were received a placebo (OR 0.26; CI 0.04, 1.23; 6 RCTs, Table 3).

Targeting specific adverse events by organ system, we found that adverse events in the neurologic/nervous system and various other organ systems (e.g., eye, ear, liver, renal, reproductive) were more likely in those taking SJW (OR 1.56; CI 1.08, 3.32; 14 RCTs); all other comparisons were not statistically significant (see Table 3). However, across studies, the adverse event assessments were limited and inadequate for the assessment of rare adverse events which lowered the quality of evidence.

### SJW vs. antidepressants

**Fig. 5:**
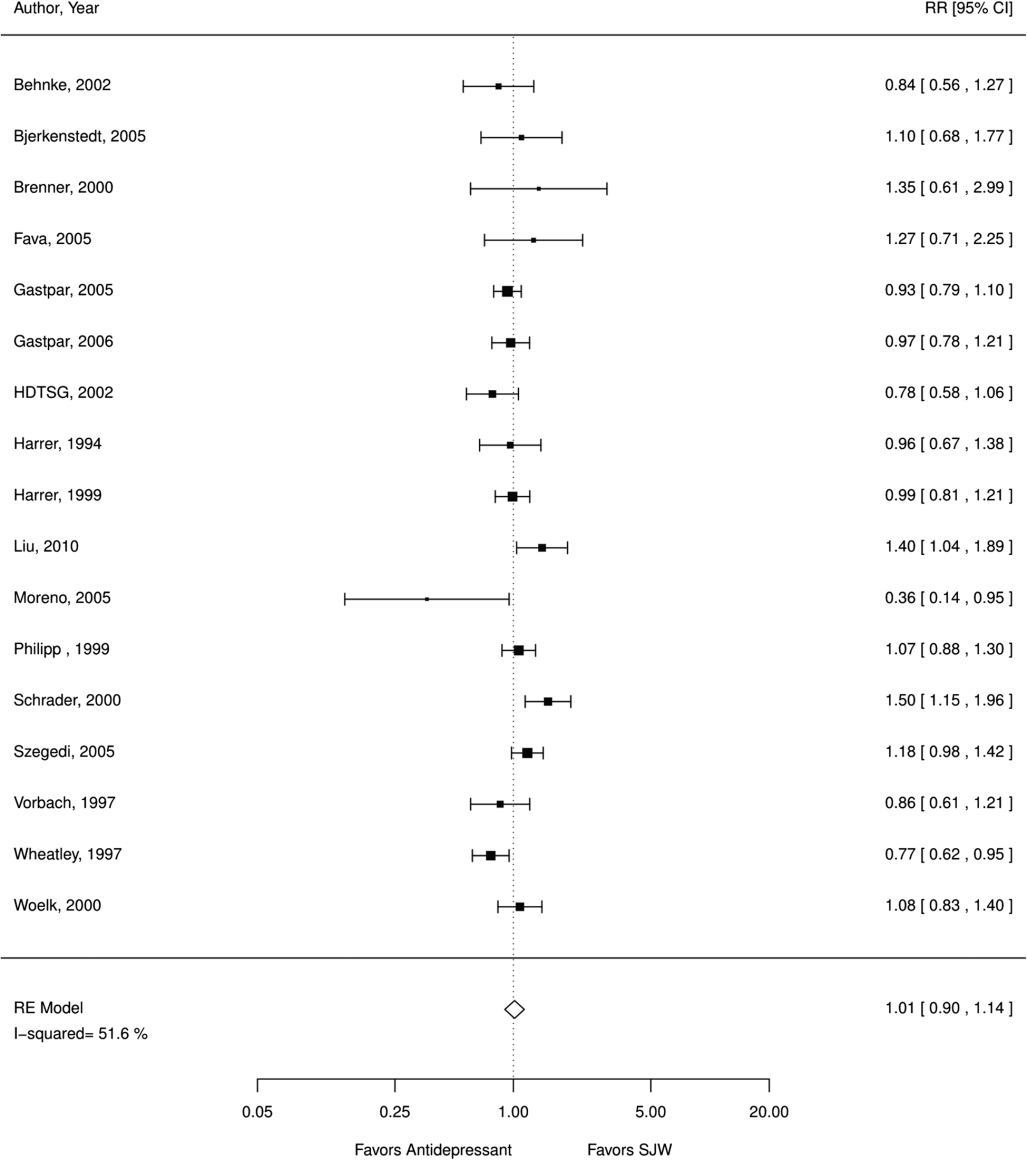
SJW vs. antidepressants, treatment responder rate; *RE* random effects, *RR* relative risk, *SJW* St. John’s wort

**Fig. 6:**
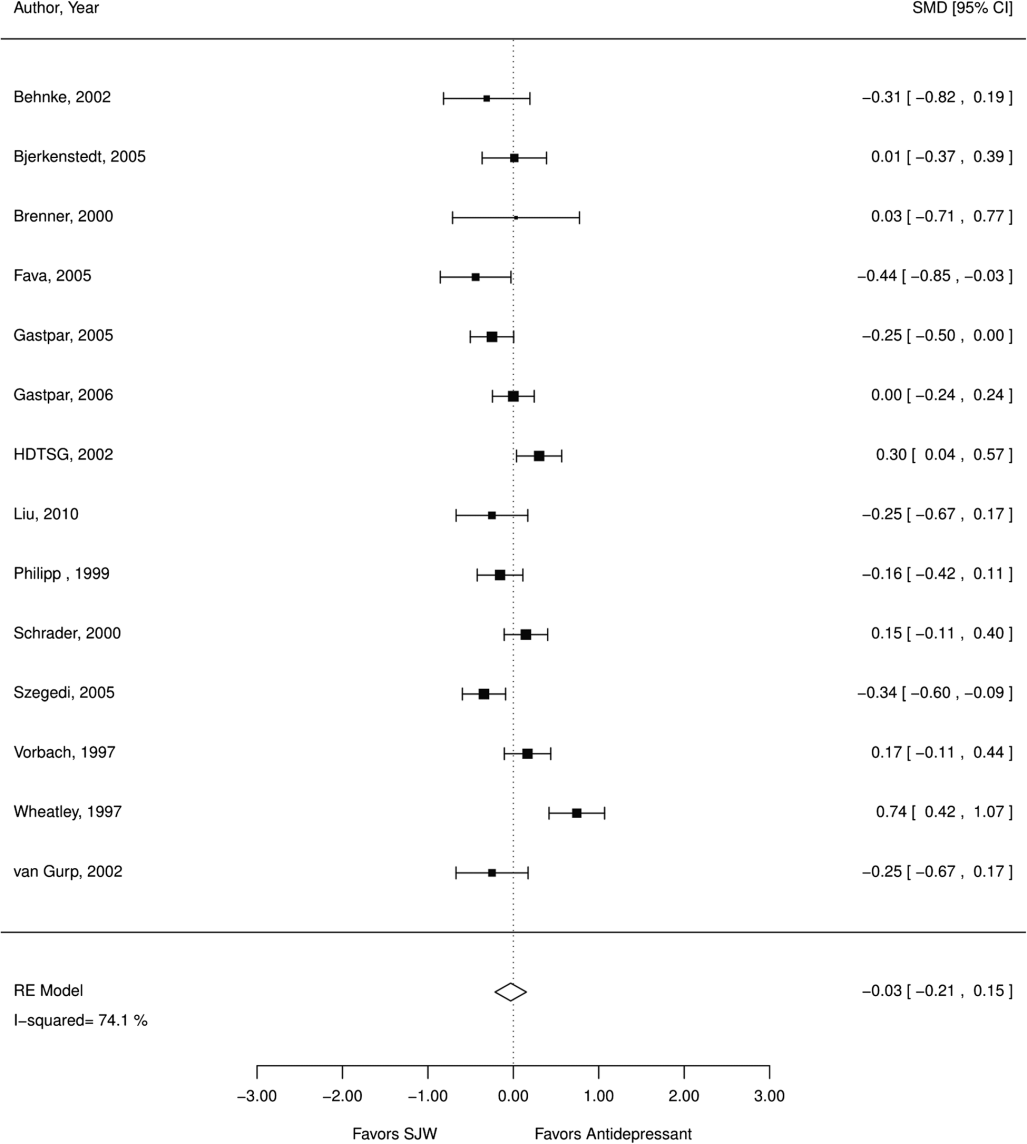
St. John’s wort vs. antidepressants, depression scale standardized mean differences; *RE* random effects, *SJW* St. John’s wort, *SMD* standardized mean differences

*a. Comparative efficacy*. The included studies showed the efficacy of SJW for depression symptoms was comparable to antidepressant medication, with SJW being neither inferior nor superior. We found no systematic differences in the rate of treatment responders (RR 1.01; CI 0.90, 1.14; 17 RCTs; I^2^ 52 %; *N* = 2776; Fig. 5) comparing SJW and standard antidepressant medication. Patients also did not have different depression scale scores (SMD −0.03; CI −0.21, 0.15; 14 RCTs; I^2^ 74 %; *N* = 2248; Fig. 6) comparing the two treatment approaches but the heterogeneity was substantial (74 %). The effects for the treatment responder rate and depression scale scores remained stable when analyses were limited to RCTs that had reported a power calculation and that had sufficient statistical power to detect differences between treatments (treatment responders: RR 0.98; CI 0.80, 1.19; 5 RCTs; I^2^ 59 %; scale scores: SMD 0.03; CI −0.75, 0.84; 4 RCTs; I^2^ 91 %). Pooled estimates were similar when excluding poor quality studies; however, the study quality of this subset of studies was limited with mostly fair quality studies, which lowered our confidence in the evidence assessment.

**Fig. 7:**
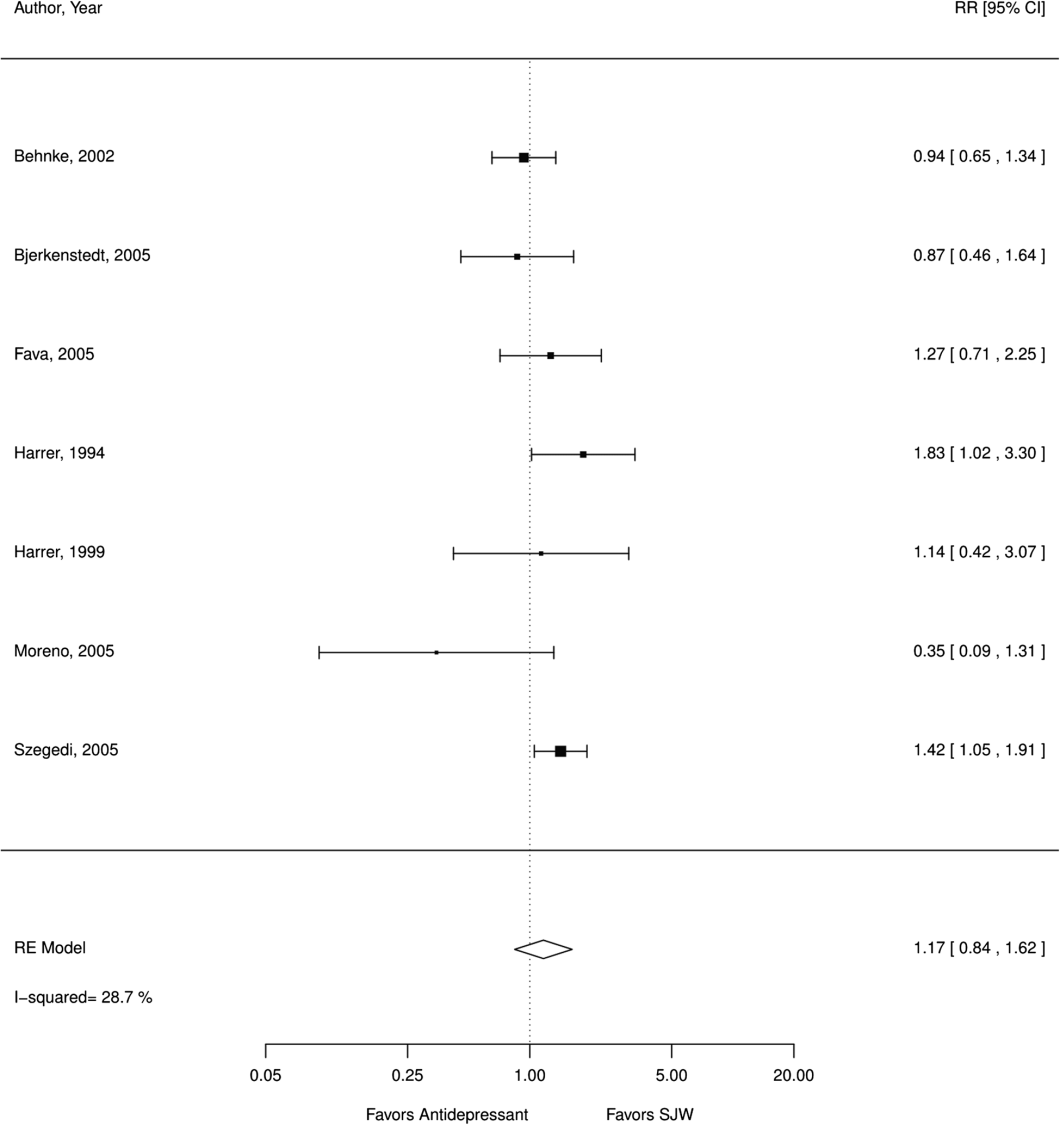
SJW vs. antidepressants, number of patients in remission; *RE* random effects, *RR* relative risk, *SJW* St. John’s wort

Patients who received SJW did not experience remission from depression at statistically significantly lower or higher rates than patients who received antidepressants (RR 1.17; CI 0.84, 1.62; 7 RCTs; I^2^ 29 %; *N* = 787; Fig. 7). However, studies reporting on remission were limited due to study quality and the statistical power to detect differences between interventions was unclear. The quality of evidence was downgraded accordingly.

Only one RCT reported on depression relapse and quality of life and effect estimates were not replicated in another, independent study resulting in a very low quality of evidence rating (Table 3).

All but one identified comparative study compared SJW to antidepressant medication. One study compared SJW and psychotherapy and no replication was identified in the literature. Meta-regressions comparing SSRIs, tricyclic antidepressants, and other antidepressants did not suggest a systematic association with the treatment effect estimates (outcome treatment responders *p* = 0.505; outcome depression scale scores *p* = 0.210; outcome remission *p* = 0.654). The majority of studies tested SJW compared to SSRIs. Subgroup analyses did not show differences between SJW and SSRIs (outcome treatment responders RR 1.02; CI 0.87, 1.20; 11 RCTs; I^2^ 52 %; outcome depression scale scores SMD 0.10; CI −0.08, 0.27; 10 RCTs; I^2^ 59 %; outcome remission RR 1.09; CI 0.76, 1.56; 6 RCTs; I^2^ 27 %), but the heterogeneity was much lower than the analyses of SJW vs. all antidepressants, indicating that the type of antidepressants may be a source of differences between study results.

*b. Comparative safety*. In the included RCTs comparing SJW to standard antidepressant medications, there was evidence that more patients taking antidepressants experienced adverse events (OR 0.67; CI 0.56, 0.81; 11 RCTs; Table 3). Specifically, SJW was associated with fewer adverse events in the gastrointestinal (OR 0.43; CI 0.34, 0.55; 15 RCTs, Table 3) and neurologic (OR 0.29; CI 0.24, 0.36; 15 RCTs, Table 3) organ systems. Adverse events involving psychiatric or sexual functioning were also lower in patients treated with SJW, but only a small number of studies reported on these symptoms. Serious adverse events did not differ statistically significantly between the treatment approaches (OR 0.62; CI 0.05, 5.46; 4 RCTs, Table 3), but this result was also based on a small number of studies.

Subgroup analyses for different types of antidepressant medication were hindered by the small number of RCTs testing a specific antidepressant and reporting on specific adverse events. In the largest group of antidepressants used in studies, SSRIs, subgroup results were similar to the main analysis, but the difference in the number of participants with adverse events was not statistically significantly different (OR 0.81; CI 0.63, 1.04; 7 RCTs). There were fewer serious adverse events in the SJW group but the difference was not statistically significant (OR 0.62; CI 0.05, 5.46; 3 RCTs) across three RCTs. In studies on tricyclic antidepressants, more participants experienced adverse events than compared to SJW (OR 0.43; CI 0.25, 0.72; 3 RCTs) but only three studies contributed to this analysis. One RCT in this subgroup that reported on serious adverse events reported the absence of events in both groups.

The rigor of adverse event assessments and the reporting of recorded events varied greatly across studies. Comparative analyses were potentially limited due to the lack of statistical power to show differences in individual rare events. In addition, the RCTs only addressed a limited range of potential adverse events. Consequently, the quality of evidence was downgraded, in particular when sensitivity analyses excluding poor quality studies could not be performed or suggested different effect estimates.

### Other results

We also investigated the comparative effects of the different extracts used in included studies. We found only one study that compared two different standardized extracts and three studies that compared different dosages, none of which found statistically significant differences between treatment arms. A meta-regression across studies did not indicate systematic differences in outcomes depending on the extract used (outcome treatment responders *p* = 0.347; outcome depression scale scores *p* = 0.127; outcome remission *p* = 0.371). An extract of 0.3 % hypericin and 1 to 4 % hyperforin was the tested extract with the largest number of RCTs (8 studies). All but one RCT evaluated SJW as monotherapy and only one RCT provided data on SJW as adjunctive therapy precluding further analyses. Although we searched the international literature without language restriction, 51 % of included studies were conducted in Germany. Meta-regressions found mixed results: no indication that effect sizes differ by study in the outcome number of responders (*p* = 0.078), number of patients with adverse events (*p* = 0.95), or the outcome depression remission (*p* = 0.058), but German studies reported a stronger effect of SJW than non-German studies for the continuous outcome change in depression rating scales (*p* = 0.012).

*Review question 2: Is there a difference in effect, depending on the type of MDD (i.e. mild, moderate, severe)?*

We examined the variation in efficacy and safety of SJW by MDD severity to answer our second review question. Of the identified studies, 12 included patients with either mild or moderate depression. Three studies are limited to patients with moderate depression alone. No study was identified that examined patients with mild depression alone. Finally, only one study was identified that focused exclusively on patients with severe depression.

### SJW vs. placebo

A meta-regression aiming to identify an association between the depression severity and the size of the treatment effect of SJW compared to placebo did not indicate a systematic difference in any of the outcomes that had sufficient study numbers to enable analyses (outcome treatment responders *p* = 0.798; outcome depression scale scores *p* = 0.365; outcome remission *p* = 0.159). We determined that the quality of evidence that suggested that there is no difference in SJW effectiveness depending on depression severity as very low (Table 3). This was due to the fact that the results were based on an indirect comparison across studies (a meta-regression), the majority of samples were in mixed patient samples of combined mild or moderate-severe depression, and the absence of data on patients with severe depression which limited the range of depression severity that was analyzed.

We also found no indication that the number of patients with adverse events differed significantly between depression severity subgroups (*p* = 0.480); however, all limitations to the evidence base outlined in the effectiveness analyses apply equally to this analysis.

The effect of SJW among only patients with mild-moderate depression was similar to main analyses for treatment responders (RR 1.45; CI 1.09, 1.92; 10 RCTs; I^2^ 71 %) and scale score (SMD 0.51; CI 0.20, 0.82; 9 RCTs; I^2^ 81 %) outcomes. Only three studies examined the effect of SJW on moderate depression against placebo, and all three showed significant effects in terms of treatment responder rate and depression scale scores [22, 37, 43]. These effects were nonsignificant in the pooled analyses of these three studies for treatment responders (RR 2.50; CI 0.16, 33.33; 3 RCTs; I^2^ 96 %) and severity (SMD 0.86; CI 1.11, 2.83; 3 RCTs; I^2^ 96 %), and we detected high heterogeneity between the trials. We identified no study reporting on patients with severe depression comparing SJW with placebo.

Analyses could only be performed for selected outcomes due to the small number of studies in some subgroups. In addition, the large majority of studies were in samples of combined mild and moderate depression, hence potentially differential effects of SJW for patients with mild, moderate, or severe depression could not be determined.

### SJW vs. antidepressants

We did not identify differences in effectiveness between the interventions in the mild and moderate subgroups analyzing the outcome number of treatment responders (RR 1; CI 0.77, 1.30; 8 RCTs; I^2^ 63 %), depression scale scores (SMD 0.16; CI 0.33, 0.65; 5 RCTs; I^2^ 76 %), or patients in remission (RR 0.89; CI 0.57, 1.41; 4 RCTs; I^2^ 0 %).

The results for the number of participants with adverse events showed similar results to the main adverse event analysis, with studies reporting fewer patients with adverse events in the SJW intervention group compared to antidepressant medication (OR 0.65; CI 0.56, 0.77; 7 RCTs).

In the subgroup of moderate depression severity, there were no differences between interventions for the outcome number of treatment responders (RR 0.98; CI 0.88, 1.09; 4 RCTs; I2 0 %) or depression scale scores (SMD 0.13; CI −0.13, 0.45; 3 RCTs; I2 4 %). One RCT in severe depression [44] reported no statistically significant difference between the SJW extract LI 160 and imipramine for the number of treatment responders (RR 0.79; CI 0.45, 1.37; 1 RCT) or mean depression scale scores (SMD −0.17; CI −0.44, 0.11; 1 RCT).

Analyses could only be performed for selected outcomes due to the small number of studies in the subgroups. In addition, studies were primarily in samples of combined mild and moderate depression severity and only one study with patient with severe depression was identified. Consequently, whether the comparison between SJW and antidepressants differs systematically by depression severity could not be determined.

## Discussion

The available evidence suggests that SJW extracts are effective in treating patients with mild and moderate MDD compared to placebo and comparable to antidepressants. Observed adverse events were fewer than compared to antidepressants, however, adverse event assessments were limited.

The existing evidence base indicates that SJW is a herbal alternative to antidepressant medication with fewer adverse events without compromising effectiveness in symptom improvement in mild and moderate depression. Improvements in depression symptoms were shown for treatment response rates and on standard clinical scales. Translating the shown effect size estimates into clinically meaningful units, the average response rate, i.e. participants showing a marked response to treatment, was 56 % for SJW compared to a response rate in patients treated with a placebo of 35 %. The mean standardized effect size estimate seen across studies is equivalent to a 3-point reduction on the HAMD scale compared to placebo treatment. Our confidence in the summary effect was downgraded to moderate quality of evidence due to heterogeneity across studies. While studies were consistently favoring SJW over placebo, the size of the treatment effect estimates varied substantially across included studies. Despite a large number of meta-regressions and subgroup analyses, we were unable to identify significant sources of differences between studies that could explain the heterogeneity shown in the pooled results. Therefore, findings have to be interpreted with caution. Future research may provide more insights for which patient group SJW is particularly effective or which intervention characteristics are associated with larger treatment effects.

Our review also addressed the outcome remission using study authors’ definitions, which usually corresponded to a HAMD score of less than seven or eight and indications that no further treatment was required. While remission rates were lower among participants using SJW compared to a placebo, these results were not statistically significant and the quality of evidence was low due to mixed study quality and differences in results across studies. The average proportion of patients in remission was 38 % in SJW treatment groups and 27 % in placebo groups.

The evidence base indicated that SJW was not less (or more) effective than antidepressants in treating major depressive disorder in patients with mild and moderate depression. Treatment response rates and depression severity did not differ between patients administered SJW and antidepressants, including studies that were explicitly designed to detect statistically significant differences between the treatment groups. Remission rates were also not significantly different but given the lack of effect shown in placebo trials and the limited quality of the identified studies this result has to be interpreted with caution. Remission rates were low in SJW as well as antidepressant arms (average 38 and 33 %, respectively); of note, the follow-up times in the included studies were relatively short (range 4–12 weeks).

Patients taking SJW were not more likely to experience adverse events than patients receiving a placebo across all assessed adverse events. Serious adverse event rates did not differ between the groups, but users of SJW experienced more adverse events related to the nervous system or to eye, ear, liver, renal, and reproductive organ systems. Conversely, SJW treatment was associated with fewer adverse events overall than antidepressants, and specifically for adverse events related to the gastrointestinal and nervous systems. Serious adverse events did not differ significantly between the two treatment groups, but only a few studies reported on adverse events and the identified RCTs were not designed to address rare adverse events. The quality of evidence of adverse event effect estimates was downgraded given that the rigor of assessments varied and the studies were not designed to detect rare events. Although all but one study reported on adverse events, the assessment and reporting varied considerably. Studies varied in particular on which adverse events they reported on; the presence or absence of serious adverse events was only addressed in a small proportion of studies. SJW has been linked to specific rare events such as hypertensive crisis and induction of mania, but the adverse event reporting in identified studies was often generic and concentrated on gastrointestinal aspects and tolerability. In order to advance our knowledge of the effects of SJW, empirical evidence of the presence and the absence of adverse events is critical and should be addressed in future research.

The presented analyses did not indicate that the effect of SJW on major depression differs by depression severity. However, the existing research is based on patients with mild or moderate depression. The mixed depression severity samples and the absence of data on patients with severe depression hindered any meaningful analysis. To date, the effects of SJW in patients with severe depression are not known. Clinicians need to be aware that results of our review may not extrapolate to include all patients with MDD.

As for clinical practice recommendations, there are demonstrated positive findings. Nonetheless, some concerns remain. Our review was in particular unable to dismiss concerns of rare adverse events that have been linked to SJW due to the lack of trials addressing these harms [7]. Some existing practice guidelines, such as the UK Guidelines for Depression in Adults [51], advise not to prescribe SJW because of uncertainty about appropriate doses, persistence of effect, variation in the nature of preparations and potential serious interactions with other drugs (including oral contraceptives, anticoagulants, and anticonvulsants). A 2012 review advised against using SJW with oral contraceptives, as well as immunosuppressants or cardiovascular drugs and a review looking specifically at warfarin found interactions between SJW and this anticoagulant [52, 53]. Furthermore, a review of popular herbal preparations found SJW interacted with more medications than any of the other herbs and dietary supplements [54]. Post-marketing surveillance of spontaneous adverse drug reactions indicated that SJW produced a similar adverse event profile to fluoxetine, with mild and severe adverse events more common with SJW while life-threatening events were more common with fluoxetine but still occurred [55]. While reports of rare adverse events cannot be dismissed based on RCT data, it is noteworthy that SJW appears to have fewer adverse events than antidepressant medication in the reported comparative analyses.

A further relevant point for practice is that the research findings are based on SJW monotherapy. Existing research used the herb SJW as an alternative treatment to antidepressant medication, not as an additional treatment option that can be added to standard treatment. This aspect is in particular relevant to patients with severe depression. Post-marketing surveillance in Australia found that, though SJW was not often given with an SSRI, there was a high proportion of adverse effects when this occurred, including a report of life-threatening serotonin syndrome [55]. While concerns about potential drug interactions will have prompted researchers to not provide patients with SJW in addition to standard antidepressant medication, we also did not identify studies that evaluated the effect of SJW treatment adjunctive to psychotherapy.

Too few studies compared the different extracts and dosages of SJW to draw meaningful conclusions about the differential effects of various types and amounts of the herb. There was similarly very low quality of evidence for the differential effect of SJW as an adjunctive therapy compared to it as a monotherapy due to a lack of trials on the comparison. The results of this review are comparable to the conclusions of a previous Cochrane review of SJW for major depression by Linde et al., in 2008, which found that SJW extracts are superior to placebo for MDD, are similarly effective as standard antidepressants, and have fewer side effects than standard antidepressants [6]. Our review included all but one of the 29 studies from that review [17–27, 29, 30, 34, 35, 37, 39–50]. One of the trials could not be retrieved [56]. Our review added an additional seven studies [16, 28, 31–33, 36, 38] that had been more recently published or included comparative effectiveness data. The proportion of non-German studies was higher in our study pool with half of included studies reporting on patients recruited in other countries. The findings of a more recent systematic review of pharmacological treatments for depressive disorders in primary care [5] were consistent with the previous review, in that hypericum extracts showed similar efficacy and better acceptability than antidepressants and are effective for the treatment of acute depression, though effects when compared to placebo were modest.

This review has several strengths: an a priori research design, a comprehensive search of electronic databases without language restriction, duplicate study selection and abstraction of study information, detailed risk of bias assessments, and comprehensive quality of evidence evaluations used to formulate review conclusions. However, some limitations are worth noting. First, we did not contact individual study authors; results reported in the review are based on published data. Some of the included studies were of poor quality, primarily due to lack of ITT or poor follow-up. The depression improvements associated with SJW were seen in the analyses of the number of treatment responders, as well as mean depression scale scores; however, both treatment effect estimates showed heterogeneity. A large number of subgroup and sensitivity analyses did not identify systematic sources of differences between studies, and heterogeneity remains as a limitation of the SJW evidence. Adverse event evidence is limited because the rigor of adverse event assessments varied greatly; comparative analyses were potentially limited due to the lack of statistical power to show differences in individual rare events; and, RCTs only assessed a limited range of potential adverse events.

Future research in this area should include more head-to-head trials between specific extracts and dosages of SJW to evaluate their comparative effectiveness. While potential risks of drug interactions hinders research of SJW as an adjunctive treatment, research on SJW concomitant to psychotherapy are also missing. Future research studies should clearly report on the presence and absence of adverse events, in particular rare events linked to SJW. As quality of life is greatly affected by MDD, it would be important to see more studies of depression treatment include this measure. Adverse events should be systematically assessed to determine concrete evidence of the presence and absence of adverse events.

## Conclusions

Our systematic review showed that SJW given as monotherapy for mild and moderate depression is superior to placebo in improving symptoms and not significantly different from antidepressant medication; however, there was evidence of substantial heterogeneity between studies and we were unable to identify systematic sources of differences between studies. In addition, there is a lack of research on applications of SJW in severe depression. SJW adverse events reported in included RCTs were comparable to placebo and fewer compared to antidepressant medication; however, adverse event assessments were limited and inadequate for rare events affecting our confidence in this conclusion.

## Additional files

Additional file 1: PRISMA 2009 Checklist; description: completed checklist of PRISMA items as required by the journal. (DOC 64 kb) Additional file 2: Search strategy; description: search terms, boolean operators, and time periods used to find literature organized by database. (DOCX 91 kb) Additional file 3: Depression scale standard cut-points; description: cut-off scores for a clinical diagnosis of depression on many validated rating scales for depression. (DOCX 83 kb)

## Abbreviations

AMED: Allied and Complementary Medicine Database
CENTRAL: Cochrane Central Register of Controlled Trials
CI: confidence interval
CINAHL: Cumulative Index to Nursing and Allied Health Literature
DSM: Diagnostic and Statistical Manual of Mental Disorders
GRADE: Grades of Recommendation, Assessment, Development and Evaluation
HAMD: Hamilton clinical rating scale for depression
ITT: intent-to-treat
MANTIS: Manual, Alternative and Natural Therapy Index System
MDD: major depressive disorder
OR: odds ratio
PICOTSS: framework of participants, interventions, comparators, outcomes, timing, settings, and study design
RCT: randomized controlled trial
RR: relative risk
SJW: St. John’s wort
SMD: standardized mean differences
SSRI: selective serotonin reuptake inhibitor
QoE: quality of evidence
USPSTF: United States Preventative Services Task Force
