# Supplementary material for: A systematic review of St. John’s wort for major depressive disorder
Source: Syst Rev. 2016 Sep 2;5(1):148. doi: 10.1186/s13643-016-0325-2 (PMC5010734; doi:10.1186/s13643-016-0325-2)
Supplement: Additional file 2: — Search strategy; description: search terms, boolean operators, and time periods used to find literature organized by database. (DOCX 91 kb) [file 13643_2016_325_MOESM2_ESM.docx]

# Additional File 2: Search Strategy

**DATABASE SEARCHED & TIME PERIOD COVERED:**

PubMed – 1/1/2007-11/24/2014

**SEARCH STRATEGY:**

"Hypericum"[Mesh] OR john's wort OR johns wort OR hyperic* OR johanniskraut

AND

"Depressive Disorder"[Mesh] OR "Depression"[Mesh] OR depress*[tiab] OR unipolar OR mood disorder* OR mood disturbance* OR affective disorder*

**==========================================================================**

**DATABASE SEARCHED & TIME PERIOD COVERED:**

CINAHL – 1/1/2007-11/24/2014

**SEARCH STRATEGY:**

john's wort OR johns wort OR hyperic* OR johanniskraut

AND

depress* OR unipolar OR mood disorder* OR mood disturbance* OR affective disorder*

**==========================================================================**

**DATABASE SEARCHED & TIME PERIOD COVERED:**

PsycINFO – 1/1/2007-11/24/2014

**SEARCH STRATEGY:**

john's wort OR johns wort OR hyperic* OR johanniskraut

AND

depress* OR unipolar OR mood disorder* OR mood disturbance* OR affective disorder*

**==========================================================================**

**DATABASE SEARCHED & TIME PERIOD COVERED:**

CENTRAL – 1/1/2007-11/24/2014

**SEARCH STRATEGY:**

john's wort or johns wort or hyperic* or johanniskraut:ti,ab,kw

AND

depress* or unipolar or mood disorder* or mood disturbance* or affective disorder*:ti,ab,kw

**==========================================================================**

**DATABASE SEARCHED & TIME PERIOD COVERED:**

Embase – 1/1/2007-11/24/2014

**SEARCH STRATEGY:**

john* NEAR/2 wort OR (johns AND wort) OR hyperic* OR johanniskraut

AND

depress* OR unipolar OR (('mood'/exp OR mood) AND disorder*) OR (('mood'/exp OR mood) AND disturbance*) OR (affective AND disorder*)

AND

Human

**==========================================================================**

**DATABASE SEARCHED & TIME PERIOD COVERED:**

AMED – 1/1/2007-11/24/2014

**SEARCH STRATEGY:**

john's wort or johns wort or hyperic* or johanniskraut

and

(depress* or unipolar or mood disorder* or mood disturbance* or affective disorder*).af.

**==========================================================================**

**DATABASE SEARCHED & TIME PERIOD COVERED:**

MANTIS – 1/1/2007-11/24/2014

**SEARCH STRATEGY:**

john's wort or johns wort or hyperic* or johanniskraut

and

(depress* or unipolar or mood disorder* or mood disturbance* or affective disorder*).af.

**==========================================================================**

**DATABASE SEARCHED & TIME PERIOD COVERED:**

Web of Science Indexes=SCI-EXPANDED, SSCI, A&HCI, CPCI-S, CPCI-SSH  –1/1/2007-1/19/2015

**SEARCH STRATEGY:**

TOPIC: (john's wort or johns wort or hyperic* or johanniskraut)

AND

TOPIC: (depress* or unipolar or mood disorder* or mood disturbance* or affective disorder*)

**==========================================================================**

**DATABASE SEARCHED & TIME PERIOD COVERED:**

ICTRP (International Clinical Trials Registry Platform–1/1/2007-1/19/2015

**SEARCH STRATEGY:**

john's wort or johns wort or hyperic* or johanniskraut

**TOTAL OF ALL RESULTS AFTER REMOVING DUPLICATES AND ANIMAL-ONLY STUDIES:** **555**
