# Supplementary material for: A systematic review of St. John’s wort for major depressive disorder
Source: Syst Rev. 2016 Sep 2;5(1):148. doi: 10.1186/s13643-016-0325-2 (PMC5010734; doi:10.1186/s13643-016-0325-2)
Supplement: Additional file 3: — Depression scale standard cut-points; description: cut-off scores for a clinical diagnosis of depression on many validated rating scales for depression. (DOCX 83 kb) [file 13643_2016_325_MOESM3_ESM.docx]

**Additional File 3: Depression Scale Standard Cut-Points**

| **Scale** | **Cut-off point** |
| --- | --- |
| Beck Depression Inventory-I | Cut-Off for Clinical Diagnosis of Depression: 0–9 = minimal/no depression 10–18 = mild/moderate depression 19–29 = moderate/severe depression 30–63 = severe depression |
| Beck Depression Inventory-II | 0-13 = minimal 14-19 = mild (13-14*= mild) 20-28 = moderate 29-63 = severe |
| CES-D | CES-D 20:  16 = “significant” or “mild” depressive symptomatology  CES-D 10:  11 = recommended as cut off (Equivalent to experiencing 6 symptoms for most of the previous week or a majority of symptoms on 1 or 2 days.) |
| Clinical Diagnosis/Meets DSM Criteria/MDI | 26 = moderate-severe depression  0-19 = no depression  20-24 = mild depression  25-29 = moderate depression  30-50 = severe depression |
| DASS-21 Depression Scale | 0-4 = Normal  5-6 = Mild  7-10 = Moderate  11-13 = Severe  14+ = Extremely severe  12 = recommended cut-point |
| Depression-Arkansas Scale (D-ARK) | 26-37= Mild  38-57 = Moderate |
| Geriatric Depression Scale (GDS) | GDS-5:  > 2 = cut-point  GDS-15: 5-9 = mild  10-15 = moderate to severe  Cut-off scores for GDS-15 Among Special Populations: Cognitive Impairment = 8 Dementia = 11 Parkinson’s Disease = 10-11 (but some variation here) Stroke = 11-12 (minor depressed)  Post Stroke = 6-7  Elderly home care = 5  GDS Long Form (30 items)  11-20 = mild  21-30 = moderate to severe |
| GHQ (General Health Questionnaire) | 4 = usual cut-point |
| Hamilton Rating Scale For Depression | 0-6 = no depression  7-17 = mild depression  18-24 = moderate depression 24+ = severe depression |
| Hospital Anxiety and Depression Scale | 0-7 = no depression  8-10 = “possible case”  11-21 = “probably case”  Optimal cutoff point = >8 for the identification of suspicious cases and >11 for safe cases on both subscales |
| MADRS-S | 13-19 = Mild  20+ = Moderate to Severe |
| MMPI Depression Scale | T score of 70 used for MMPI T score of 65 used for MMPI-2 |
| Montgomery-Asberg Depression Rating Scale (MADRS) | 7-19 = Mild  20-34 = Moderate  35-60 = Severe |
| MOS-D (Medical Outcomes Study Depression Screen) | 0.06 = usual cut-point |
| PHQ-9 | 5 = mild  10 = moderate  15 = severe  *10 cited as the optimal cut off point |
| PRIME-MD (Primary Care Evaluation of Mental Disorders) | 1 = usual cut-point |
| SCL-20 | ≥1.75 as a cutoff for major depression |
| SCL-CD6 | ≥17 is indicative of MDD |
| SDDS-PC (Symptom Driven Diagnostic System-Primary Care) | 2 = usual cut-point |
| Zung SDS (Zung Self Assessment Depression Scale) | 50 = mild  60 = moderate  70 = severe |
| IPAT | N/A |
| PROMIS Depression | N/A |
| SCL-90 | N/A |
| Brief Symptom Inventory (BSI) | N/A |
| Alasker scale | N/A |

Note: N/A: Not applicable; Based on Sorbero et al. (unpublished), Forces and Resources Policy Center of the RAND National Defense Research Institute, RAND Corporation, 2013
